# Induction of diploid gynogenesis in an evolutionary model organism, the three-spined stickleback (*Gasterosteus aculeatus)*

**DOI:** 10.1186/1471-213X-11-55

**Published:** 2011-09-12

**Authors:** Irene E Samonte-Padilla, Christophe Eizaguirre, Jörn P Scharsack, Tobias L Lenz, Manfred Milinski

**Affiliations:** 1 Department of Evolutionary Ecology, Max-Planck Institute of Evolutionary Biology, August-Thienemann Str 2, Ploen 24306, Germany; 2 Leibniz Institute for Marine Sciences (IFM GEOMAR), Evolutionary Ecology of Marine Fishes, Dusternbrooker Weg 20, 24105, Kiel, Germany; 3 Department of Animal Evolutionary Ecology, Institute for Evolution and Biodiversity, WWU Muenster, Huefferstrasse 1, 48149 Muenster, Germany

## Abstract

**Background:**

Rapid advances in genomics have provided nearly complete genome sequences for many different species. However, no matter how the sequencing technology has improved, natural genetic polymorphism complicates the production of high quality reference genomes. To address this problem, researchers have tried using artificial modes of genome manipulation such as gynogenesis for fast production of inbred lines.

**Results:**

Here, we present the first successful induction of diploid gynogenesis in an evolutionary model system, the three-spined sticklebacks (*Gasterosteus aculeatus*), using a combination of UV-irradiation of the sperm and heat shock (HS) of the resulting embryo to inhibit the second meiotic division. Optimal UV irradiation of the sperm was established by exposing stickleback sperm to a UV- light source at various times. Heat shock parameters like temperature, duration, and time of initiation were tested by subjecting eggs fertilized with UV inactivated sperm 5, 10, 15, 20, 25, or 30 minutes post fertilization (mpf) to 30°C, 34°C, or 38°C for 2, 4, 6 or 8 minutes. Gynogen yield was highest when stickleback eggs were activated with 2 minutes UV-irradiated sperm and received HS 5 mpf at 34°C for 4 minutes.

**Conclusions:**

Diploid gynogenesis has been successfully performed in three-spined stickleback. This has been confirmed by microsatellite DNA analysis which revealed exclusively maternal inheritance in all gynogenetic fry tested. Ploidy verification by flow cytometry showed that gynogenetic embryos/larvae exhibiting abnormalities were haploids and those that developed normally were diploids, i.e., double haploids that can be raised until adult size.

## Background

The genomics community has been enormously revolutionized by first- and next generation sequencing (NGS) technologies [1-3] that generated vast amounts of genetic data ranging from unicellular (bacteria) to multicellular (eukaryotic) genomes. However, no matter how good these technologies are, polymorphisms in a genome complicate the assembly process, results in lower quality, and the contiguity and completeness of assembly is significantly lower than would be expected from a homozygous template [4]. Hence, there is a growing interest for the development of inbred lines, such as haploid and double haploid (DH) lines, which are particularly advantageous in genomics [5,6] because of their homozygosity and their growth potential. Inbred lines are also useful in physical mapping [7] and in genetic mapping [8] which would enable precise positioning of the genes in the genome, and thus facilitate accurate identification of candidate genes. Furthermore, DHs are also useful for mutagenesis and genetic transformation studies [9-14].

An inbred line is normally produced by classical sibling mating which is not only time and space consuming, but also laborious and expensive, especially in organisms with long generation times. Alternatively, it can be produced via androgenesis or gynogenesis which both requires inactivation of the genetic material through chemical means or by ionizing radiations. For species with relatively large eggs (1.5 - 1.7 mm), with thick membrane that hardens upon contact with water and contains numerous oil globules like that of the three-spined stickleback, optimum inactivation of the genetic material of the eggs is difficult and hence gynogenesis should be favored. Gynogenesis is defined by Thorgaard [15] as an all maternal type of inheritance wherein the genetic material of the sperm does not contribute to the resulting embryo. This process occurs naturally in some fish species like in crucian carp and in several species of poecilids [16-18]. Induction of diploid gynogenesis involves egg activation by irradiated homologous or heterologous sperm, and diploidization by retention of the second polar body (meiotic gynogenesis) or suppression of the first mitotic cleavage (mitotic gynogenesis) [19] using strong physical treatments (i.e., shocks). The most commonly used treatments are either low or high temperatures (cold or heat shocks) or hydrostatic pressure. Under the influence of such treatments, the spindle fibers are destroyed and cell division stops resulting in the fusion of the daughter cells, thus forming diploid gynogenetic embryos. Mitotic gynogenesis was regarded as the fastest way to produce DH [completely inbred (homozygous) fish]. It requires only one round of gynogenetic manipulation. Production of DHs via meiotic gynogenesis, on the other hand, requires an additional 2-3 rounds of gynogenetic manipulations. Prior to the boom of the genomics era, artificial gynogenesis has already been applied in many fish species mainly because of its potential value in experimental genetics and aquaculture [20,21]. Furthermore, it has played an important role in fish genetic improvement and control of reproduction [20]. For instance, diploid gynogenetic fish were observed to grow faster and have stronger resistance to disease than haploids [22]. Successful gynogenetic manipulations were done, e.g., in rainbow trout [20], medaka [23], common carp [24], muskellunge [25,26], goldfish [27], Russian sturgeon and starlet [28], mud loach [29], sea lamprey [30], and in Wels- and channel catfish [6,31].

The three-spined stickleback is a teleost that exhibits multiple examples of parallel evolution. It inhabits different marine, estuarine, and freshwater systems all over the Northern hemisphere and is characterized by rapid and repeated ecological and phenotypic divergence from the marine ancestor to the freshwater forms [32,33]. Like other model systems, the linkage and chromosome maps of this fish have already been published [34,35] and its genome sequenced [36]. Genomic information has now been used to understand many fundamental evolutionary problems. For instance, the successful elucidation of the evolution of body armor reduction in three-spined sticklebacks from the phenotypic down to the gene level [37] prompted Gibson [38] to elevate the status of this species to an evolutionary supermodel. The advent of NGS has also advanced studies on the population genomics of this species [39]. However despite all these developments, the problem with polymorphism remains and left a significant part of the genome unassembled. Thus, increasing the need for the production of an inbred three-spined stickleback.

In our knowledge, no inbred line is available in this species partly because the emphasis has always been on the study of natural populations. Information about effective modification of genotype in this species was limited only to ploidy manipulation using temperature shock that resulted in the production of triploid and haploid individuals [40,41]. These haploids would be ideal for genomics but as in many attempts of fish haploid production, these haploids survived only until embryonic development [20,42-46] or right after hatching [10,47,48] and hence do not provide enough template material for downstream processing.

Here, we present a working protocol for the induction of gynogenesis using a combination of sperm UV irradiation and heat shock (HS) treatment. This study aims at producing inbred lines of the three-spined stickleback suited not only for whole genome sequencing, physical and genetic mapping but also for future mutagenesis and experimental studies. Success of gynogenesis is confirmed by microsatellite DNA analysis and the ploidy of the gynogenetic embryos/larvae is verified by flow cytometry.

## Results

### Screening for the optimum UV irradiation exposure

Optimum UV irradiation of the sperms was done by exposing groups of sperms to the UV light source at various durations. UV irradiation of the sperms worked well for both 2 min and 4 min exposure with fertilization rates of 97% and 93%, respectively (Table 1). All the embryos, however, developed abnormally and did not hatch. They were collected at day 3 or 4 for DNA extraction. The sperms irradiated for 6 and 8 minutes failed to activate the stickleback eggs, thus no embryo was produced. This suggests that long exposure to UV irradiation could already be lethal to three-spined stickleback sperms as evidenced by the presence of non-motile sperms after irradiation in these treatment groups.

**Table 1:** Embryonic survival of three-spined sticklebacks subjected to UV irradiation at 4 various times resulting to zero fertilization success

Length of UV Irradiation (min)	Number of eggs	Fertilization rate (%)	Hatched fry (%)
0	76	98	98
2	74	97	0
4	80	93	0
6	67	0	0
8	67	0	0

The normal controls, on the other hand, were not only successfully fertilized but also have high percentage of survival to the eyed stage (98%) and hatched as expected at day 6 or 7.

### Effects of heat shock on chromosome diploidization of the activated stickleback eggs

The effectiveness of heat shock (HS) in promoting diploid gynogenesis, expressed by the number of gynogens produced (total gynogen yield), was clearly shown in two separate experiments. Gynogen yield was computed as the percentage of hatched, surviving fry over the total number of fertilized embryos.

**Figure 1:**
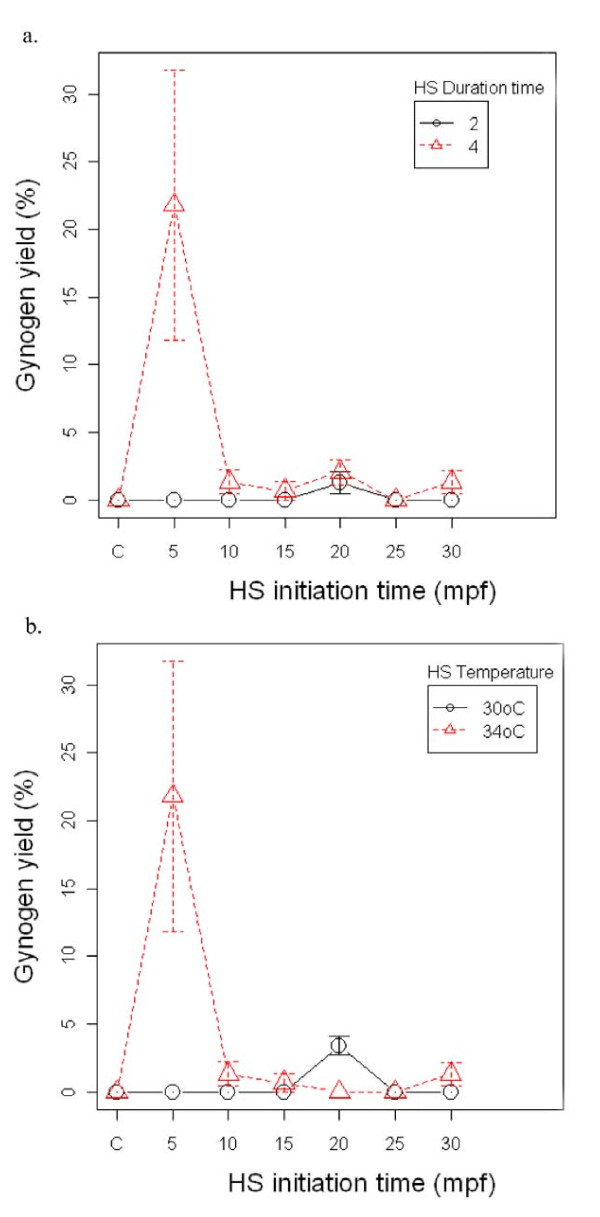
**Effects of the initiation time of heat shock (HS) on diploid gynogen production**. Eggs activated with UV irradiated sperms at different initiation times were heat-shocked at two different temperatures (30°C and 34°C) for 2 and 4 minutes. Control (C) served as HS control activated with UV irradiated sperms but without HS treatment. Effects of HS initiation time on diploid gynogen yield in terms of HS duration (Figure 1a) and in terms of HS temperature (Figure 1b) were showed. Here, results were presented as means and standard error of the raw data from three independent trials.

In the first experiment, the optimal initiation time of heat shock was established to be at 5 minutes post fertilization (mpf) in combination with heat shock at 34°C for 4 minutes. This treatment group produced the highest diploid gynogen yield of 22% (Figure 1). A low gynogen yield of 3% was also observed in the treatment group subjected to 30°C applied 20 mpf (Figure 1b). The rest of the treatments produced low yields of diploid gynogens (0-2%).

**Figure 2:**
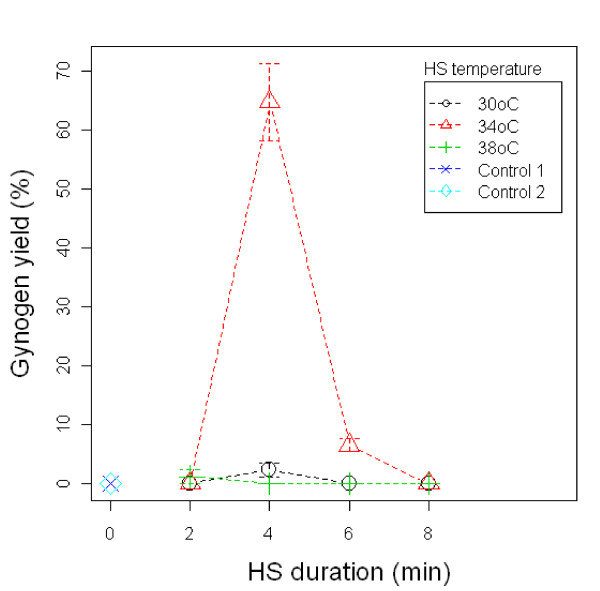
**Effects of temperature and duration of HS on diploid gynogen yield**. Eggs activated with UV irradiated sperm were heat shocked starting at 5 mpf at 30°C and 34°C for 2, 4, 6, and 8 minutes. Control 1 and Control 2 served as HS controls activated with UV irradiated sperms 5 mpf and after all the UV treatments, respectively, but not heat shocked. Here, results were presented as means and standard error of the raw data from three independent trials.

Using the previously established HS initiation time of 5 mpf and UV irradiation of 2 min, optimal temperature and duration for applying HS was found to be at 34°C for 4 minutes. This treatment group produced a much improved gynogen yield of 65% (Figure 2) while the remaining groups that were heat shocked at 30°C and 38°C for shorter (2 minutes) and longer (>4 minutes) both yielded low diploid gynogenetic fry (<5%). In addition to HS initiation time (*p *= 0.023) and HS duration (*p *= 0.003), HS temperature (*p *= 0.001) was also found to contribute statistically significant effects on the induction of diploid gynogenesis in three-spined stickleback.

All embryos in the HS controls developed into abnormal embryos/larvae which indicated that UV irradiation for 2 min successfully inactivated the genetic material of the sperms but retained the ability to induce the development of the eggs. Such haploid (n) eggs develop abnormally in the absence of HS treatment but become morphologically normal (2n) when optimal HS condition was applied to them.

### Microsatellite DNA analysis of gynogenetic families

**Figure 3:**
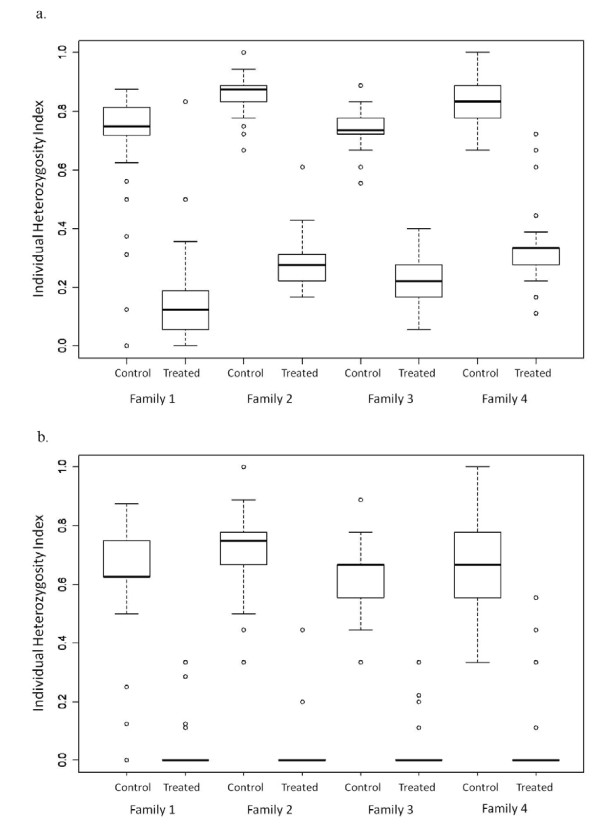
**Heterozygosity of gynogenetic families revealed by microsatellite DNA data**. Nine microsatellite loci were used to analyse the heterozygosity of four gynogenetic families (Treated Family 1-4) and their corresponding positive controls (Control Family 1-4, no UV irradiation and no HS) were analysed using the whole data set (Figure 3a) and by excluding individuals homozygous for alleles shared by the two parental fish and hence scored as "0.5" (Figure 3b).

Using the here established optimal condition for stickleback diploid gynogenesis induction, additional six gynogenetic families designated Family 1-6 were produced for microsatellite analyses. In families 1 - 4, all gynogenetic embryos/larvae were collected at day 7 and used to assess level of heterozygosity in the gynogenetic individuals. Families 5 and 6 were used to determine the type of gynogenesis that was induced in this experiment, hence only the embryos/larvae that hatched and survived past 120 days were collected and tested. In general, all the gynogenetic embryos/larvae subjected to microsatellite analysis using 9 loci were found to carry only the maternal alleles and as expected all the normal controls had biparental genotypes. Data from the four gynogenetic families produced to assess genetic homogeneity of the resulting gynogens revealed that families 1 to 4 had statistically significant lower heterozygosity index values compared to normal control fish (*p*<0.05). As shown in Figure 3, the microsatellite data were analyzed in two ways: 1) by using the whole dataset and 2) by excluding individuals homozygous for allele shared by both parents (scored as "0.5"). In both cases, gynogens were found to be more homozygous than the control fish. The degree of individual homozygosity, though, was further intensified when alleles shared by both parents were excluded in the test (Figure 3b).

Moreover, in families 5 and 6 (Table 2), 97-100% of the samples were heterozygous for the maternal alleles. This suggests that majority of the gynogens are meiotic diploids produced by retention of the second polar body. The remaining 3% found to be homozygous for the maternal alleles could be mitotic gynogens produced by inhibiting first mitotic division. These embryos can be good candidate for a completely homozygous inbred fish.

**Table 2:** Summary of microsatellite genotyping data of the two gynogenetic families used to assess the mode of gynogenesis occurring in three-spined stickleback

	Number of embryos			Number (%) of surviving	
Family	Fertilized	Abnormal	120 days old	homozygote	heterozygote
Family 5	86	20	66	2 (3.03)	64 (96.07)
Family 6	53	13	40	0 (0)	40 (100)

### Ploidy level of gynogenetic fry

**Figure 4:**
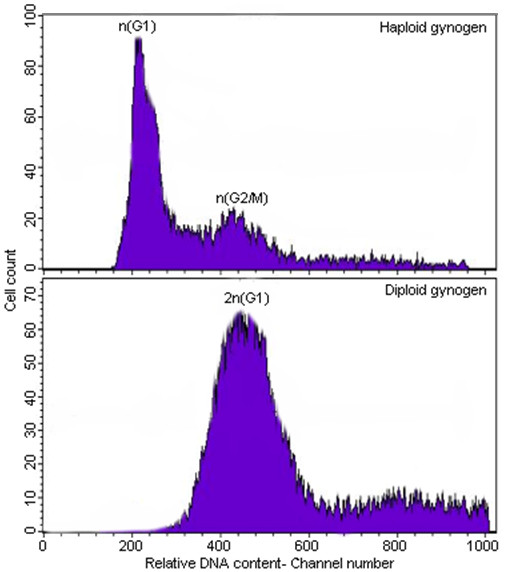
**Representative fluorescent distribution of cells prepared from gynogenetic three-spined stickleback embryos measured by flow cytometry**. The mean fluorescence peaks of representative haploid and diploid gynogenetic embryos/larvae have mean fluorescence at mean channel numbers 230 and 460, respectively.

A total of 145 gynogenetic and 16 normal control embryos/larvae were subjected to flow cytometry for relative DNA quantification. Of the 145 gynogenetic embryos/larvae, 102 exhibited abnormal features while the remaining 43 were morphologically normal. A histogram of the relative DNA content of a haploid (n) embryo is illustrated in Figure 4. It had a dominant peak at mean channel 230 which indicates that most of the cells are in the n(G1) phase and a minor peak at mean channel 460 representing cells in the n(G2/M) phase of the cell cycle. Also shown is a histogram of a diploid embryo characterized by a prominent peak at channel 460. The ploidy of the sample is reflected by the dominant peak suggesting that abnormal embryos are haploid (1n, mean channel 230) and the morphologically normal gynogens are diploid (2n, mean channel 460) with twice as much genetic material as the latter.

### Development of the gynogenetic embryos/larvae

**Figure 5:**
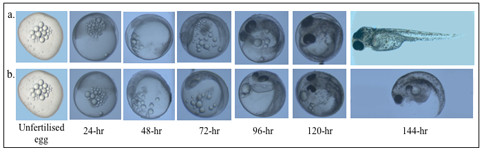
**Embryologic development of representative gynogenetic three-spined stickleback embryos**. Development of the embryos leading to a normal (Figure 5a) and abnormal (Figure 5b) gynogenetic larvae after receiving HS treatment 15 mpf for 4 min at 34°C were shown.

**Figure 6:**
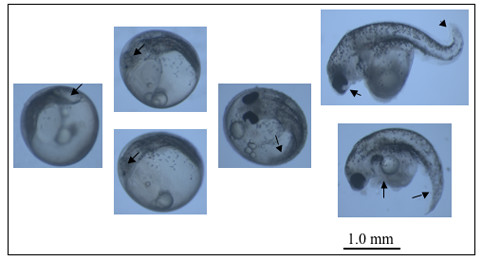
**Commonly observed abnormalities in gynogenetic three-spined stickleback embryos**. Abnormal cell proliferations (marked by black arrows) were commonly found in abnormal gynogenetic three-spined sticklebacks. These embryos often died within the first week.

The daily larval development of representative stickleback embryos activated with UV-irradiated sperm and with further HS treatment at 34°C for 4 minutes 5 mpf was monitored for one week (Figure 5). The diploid embryo in Figure 5a followed normal development as described by Kuntz & Radcliffe [49]. However the growth of the haploid embryo started to slow down 48 hours after fertilization. It managed to cover only one half of the circumference of the yolk sac and growth from here on was restricted horizontally increasing the volume of the fish but not its length (Figure 5b). The diploid embryos hatched at day 6 or 7 post fertilization, whereas haploid embryos did not hatch and remained within their enveloping membrane until they die. Death of the haploid embryo could be attributed to the many deformities observed mainly in the head and tail regions, as well as in the abdomen where the yolk sac is attached (Figure 6). Tissue degeneration at the abdominal region prevents absorption of the yolk sac thereby cutting the source of nutrients of the developing embryo leading to the death of the embryo.

## Discussion

Gynogenesis is regarded as a powerful tool in the production of an all female progeny, in studying mechanisms of sex determination, and in the production of completely inbred lines. Inbred lines are useful for whole genome sequencing and for production of mutational and developmental genetic screens. For an evolutionary model organism like the three-spined stickleback, optimization of this method will be immensely important.

In this study, UV irradiation of the sperm and heat shock (HS) of the resulting embryos were used to induce gynogenesis. UV irradiation was used because aside from being effective in deactivating the DNA of the sperm, it is cheap and easy to set-up as compared to ionizing irradiations which are more dangerous and require special containment area [20,21]. Since DNA irradiation is a key step to successful gynogenesis, it is therefore important to determine optimal condition for sperm irradiation. UV irradiation should inactivate the sperm DNA maximally and avoids chromosome fragmentation, because partially inactivated sperm DNA interferes in the embryonic development whereas over-exposure to UV light may already kill the sperm [19]. In this study, 2 to 4 minutes exposure to UV irradiation can be regarded as optimal with fertilization success of 93-97%. All the resulting embryos were morphological abnormal (Figure 5b and 6), confirmed to be haploids by flow cytometry, and had only the maternal genotype. However, UV exposure of more than 4 min in our case was already lethal to the sperms resulting in zero fertilization success. This was also indicated by the observed absence of sperm motility in these treatment groups after UV exposure. Although both 2 and 4 min UV irradiations were successful in inactivating the genetic material of the sperm, we used the former in the succeeding experiments to minimize possible "Hertwig effect" wherein haploid embryos resulting from the total destruction of the genetic material in the sperm by high doses of radiation would survive longer than those exposed to lower UV dosage [50,51].

Application of HS was observed to be effective for the retention of the second polar body in eggs activated with UV irradiated sperms resulting in morphologically normal gynogenetic diploid larvae [25,27,52-54]. For HS to be successful, right combination of the different parameters namely temperature, duration, and initiation time of application is critical. From the review of Komen & Thorgaard [21], it is suggested that optimal temperature for HS is species-specific and could vary from 30°C to up to 42°C. Since HS acts through depolymerization of protein complexes, then the best temperature should be close to the upper limit of tolerance. Likewise, optimal time for applying heat appears to be slightly different [55,56]. Timing of HS is very crucial as this decides the kind of gynogens produced. Generally, when HS is applied early, it targets second meiotic division leading to the retention of the second polar body, thereby producing diploid meiotic gynogens. Late application of heat treatment, on the other hand, results in suppression of the first mitotic cleavage, producing an all homozygote female fry (inbred clone). Here, the best yield of diploid gynogens (65%) was observed in eggs activated with UV irradiated sperm and heat shocked at 34°C 5 minutes post fertilization (mpf) for 4 minutes (Figure 2). Although aiming at different end products, the optimal temperature and duration of HS established here correspond to what was used by Swarup [40,41] in his first attempt to produce haploid and triploid three-spined sticklebacks without UV irradiation. Independently tested, this clearly shows that HS at 34°C for 4 minutes can effectively destabilize and disorganize the microtubules of activated stickleback egg, thereby facilitating diploid gynogenesis. The comparatively low diploid gynogen yields at lower (30°C) and higher (38°C) temperatures indicates that 30°C can still be well tolerated by the developing embryo. The effect of temperature shock at 30°C is very minimal, whereas shock at 38°C could have already interfered in a wider range of developmental mechanisms resulting to further abnormalities that drives the embryos to death. Like in common carp [53], the present study clearly demonstrated that temperature, duration, and initiation time of HS play an important role in the success of diploid gynogenesis in three-spined sticklebacks.

Success of gynogenesis induction in three-spined stickleback is verified by microsatellite analysis. Microsatellite DNA technology is commonly used because of its PCR-based, co-dominant Mendelian inheritance, and highly polymorphic characteristics which facilitate molecular typing of the gynogenetic embryos/larvae [57,58]. Success is measured by the absence of paternal alleles in the resulting gynogenetic individuals. In the six families tested here, it was clear that the paternal alleles are absent and only the maternal alleles are passed on to the gynogenetic embryos/larvae. The predominance of heterozygotes (97%) for the maternal alleles only suggests that the established optimum HS condition promotes retention of the second polar body, thereby producing mostly heterozygous meiotic diploid gynogens which have the expected high degree of homozygosity (Figure 3). With the relatively early timing of heat shock (5 mpf) and due to the mode of egg collection (mainly hand stripping), it could be that the eggs used in this study are still undergoing meiosis when the HS was applied. Flow cytometry remains the best method for ploidy determination in gynogenetic stickleback. This method clearly shows that stickleback gynogenetic embryos/larvae that developed normally were diploids (2n) whereas those that showed abnormalities were haploids (n) with peaks at mean channel numbers 460 and 230, respectively (Figure 4). We observed no mosaic or aneuploid, chromosomal anomalies that are also reliably detected through flow cytometry. With the accuracy of the result obtained here, we find no reason to use more laborious and time consuming classical methods of ploidy determination like chromosome preparation and silver staining of nuclear organizer regions (NORs). Since in the present study, possible effects of doublets on fluorescence were excluded in all samples, the observed predominance of haploid cells at n(G1) phase with a significant 5% of the cells staying at the n(G2/M) is quite striking (Figure 4a). This is comparable to what was observed in muskellunge by Lin and Dabrowski [26] which suggests that haploid cells have an abnormal cell cycle that probably takes longer in time than that of their diploid counterparts. This could also mean production of lower cell numbers, hence the observed stunted growth of haploids.

## Conclusions

In summary, a working protocol for the induction of diploid meiotic gynogenesis in three-spined sticklebacks is presented in this study. The optimal condition for the inactivation of the sperm and the HS treatment to effectively suppress the second meiotic division in stickleback eggs were established to be at 2 minutes UV irradiation and HS at 34°C 15 mpf for 4 minutes, respectively. Flow cytometry and microsatellites analysis proved to be an ideal combination of methods to determine the ploidy and the all maternal inheritance in the gynogenetic individuals produced.

## Methods

Sticklebacks were collected from Grosse Plöner See (northern Germany) from November 2009 to February 2010. The fish were acclimatized first for 3 months in winter (4°C), another 3 months in spring (12°C), before finally being transferred to individual tanks and placed in summer condition (18°C) until they became reproductively mature. Stickleback males with conspicuously developed red-orange coloration on their throat region and gravid females with extended belly and dilated cloacal opening ready to spawn were used in this study.

Male fish was sacrificed by decapitation. The testes were carefully dissected out, and placed in a 40 μm cell strainer (Falcon, BD Biosciences, Europe) standing inside a small plastic petri-dish containing appropriate amount of Hank's Balanced Salt Solution (HbSS, Sigma). Using a mini pestle, the testes were carefully pressed against the mesh of the strainer to release all the sperms into the HbSS. The motility and relative quantity of the collected sperm was checked under the microscope. Only sperm solutions with active and motile sperms were used in the experiments. Likewise the gravid stickleback female was either sacrificed by decapitation and the ovaries were carefully dissected or hand stripped to collect the eggs. The latter method is advantageous because a female can be used several times.

### Sperm Inactivation by UV Irradiation

Successful irradiation means finding the adequate radiation that will result to total genetic inactivation of the sperm without destroying their ability to initiate fertilization. To attain this, we first determined the appropriate duration of UV irradiation. The experiment was done twice and each consisted of two males and five female sticklebacks. Sperm from two male sticklebacks were pooled into a 2-ml sperm suspension which was further divided into five parts: one part was not exposed to UV irradiation and served as control. The other four parts were exposed to a UV light source for to 2, 4, 6, and 8 minutes irradiation, respectively. To ensure optimum irradiation, the sperm suspension was placed on thin watch glass and irradiated at a distance of 30 cm under a 280 ultra-violet (type C) light source with continuous gentle shaking at 20 rpm. The sperm was then used to fertilize groups of pooled eggs (58-93 eggs per group) 5 minutes after dilution and UV irradiation. Fertilization was terminated by addition of 7.5 ml hatchery water, which induces swelling of the egg and closure of the micropyle. Activated eggs were transferred to well-aerated breeding jars and grown at 18°C for 6 to 7 days. The breeding jars were kept in the dark in the first 24 hours to avoid possible photo-repair of the UV-inactivated sperm DNA. The number of fertilized and unfertilized eggs was counted on day 2. Unfertilized eggs were removed immediately to avoid fungal contamination and the embryos were allowed to develop until day 6 or 7. Survival to the eyed stage and hatching was observed and recorded at day 7.

### Heat Shock (HS) Treatment to Promote DNA diploidization

Two experiments were conducted to determine the effects of HS on the diploidization of the stickleback egg activated with UV irradiated sperm. In both experiments, stickleback gametes were prepared as described above. Each of the experiments was repeated three times using independent sets of females allowing the effect of each treatment to be tested statistically. Since three-spined sticklebacks normally produce about 150-200 eggs at a time, the pooling of gametes strategy of Lin and Dabrowski [26] was adopted to enable us to investigate a wider range of combinations of HS treatments. All UV irradiations from here on were performed for 2 min.

The first experiment was designed to estimate the effect of initial time of HS on chromosome diploidization. The properly mixed pool of stickleback eggs was divided into 25 groups, each containing around 20-35 eggs. One group served as HS control composed of eggs fertilized with UV-irradiated sperms but not heat-shocked. The other 24 groups were subjected to HS in a full factorial design at 30°C or 34°C regulated water baths for 2 or 4 minutes 5, 10, 15, 20, 25, or 30 mpf. To ensure homogeneous water temperature, the water bath was kept shaking at 30 rpm. A second experiment was intended to further explore the optimal level of temperature and HS duration. The pooled eggs were divided into 14 groups. Two groups that were not heat shocked but were fertilized with irradiated sperms served as controls: HS Control 1 was transferred to the breeding jar immediately 5 mpf whereas HS Control 2 was allowed to continue fertilization until all the HS treatments were finished. The remaining groups were heat-shocked at three different temperatures (30, 34, and 38°C) for 2, 4, 6, or 8 minutes, respectively. Breeding proceeded as previously described. Like in the UV irradiation experiments, the number of fertilized and unfertilized eggs was recorded on day 2. Unfertilized eggs were removed and the fertilized embryos were allowed to grow up to day 6 or 7 after which the embryos were collected and sorted into two groups based on their morphological features: normal embryos that hatched and exhibited normal development and abnormal embryos showing haploid syndromes.

Using the here established optimal condition for stickleback diploid gynogenesis induction, additional six gynogenetic families designated Family 1-6 were produced for microsatellite analyses. In families 1 - 4, all gynogenetic embryos/larvae were collected at day 7 and used to assess level of heterozygosity in the gynogenetic individuals. Families 5 and 6 were used to determine the type of gynogenesis that was induced in this experiment, hence only the embryos/larvae that hatched and survived past 120 days were collected and tested.

### Molecular Analyses

#### Genomic DNA isolation for microsatellite DNA analysis

Genomic DNA from 4-7 day old whole embryos or caudal fin clips from the surviving gynogenetic fry and stickleback parents was extracted using Qiagen DNAeasy Kit (Hilden, Germany). The quantity of the extracted DNA was measured using Nanodrop (PeqLab Biotechnology GmbH, Germany). A total of 9 microsatellites multiplexed in 2 polymerase chain reaction (PCR) protocols [59] were used to confirm the exclusively maternal mode of inheritance of the resulting gynogenetic embryos and fry.

#### Ploidy determination by flow cytometry

Relative DNA content of embryonic/larval cells was used to infer ploidy of the gynogenetic stickleback embryos/fry. DNA content of cells was measured after DNA staining with propidium iodide by means of flow cytometry (FACS Calibur, BD Biosciences, Belgium) according to the combined protocols used for ploidy determination [60] and for cell cycle analysis of stickleback leukocytes [61] with some modifications. In brief, embryos and fry were collected between 11:00 to 13:00 and individually placed in a 40 μm cell strainer (Falcon, BD Biosciences, Europe) standing inside a small plastic Petri-dish containing 1 ml RPMI 1640 medium (Sigma) with 10% v/v distilled water (R-90). Single cell suspensions from embryos/larvae were produced by pressing it through the nylon mesh of the strainer with the help of a pestle. The resulting cell suspensions were transferred to individual wells of a 96 well deep-well micro titer plate and washed twice (600 × g, 10 min, 4°C) with 0.8 ml cold R-90 medium. After washing, cells were resuspended with 200 μl R-90 and fixed with 800 μl ice cold 99% ethanol. After fixation, plates were centrifuged (600 × g, 10 min, 4°C), the supernatant ethanol was removed and the cells were resuspended with 250 μl Sheath fluid (BD) containing RNAse (0.5 mg L^-1^) to remove background staining of RNA. The cell suspension was incubated for 10 minutes at room temperature before it was stained with propidium iodide (0.5 mg L^-1^, Sigma), a red fluorescence dye which intercalates with double stranded DNA. Red fluorescence characteristics of at least 10,000 cells were recorded per sample by means of flow cytometry. For analysis, doublet cells were subtracted from single cells as described by Wersto and co-workers [62]. DNA content of single cells was analyzed by CellQuest (BD Immunocytometry Systems, San Jose, CA).

### Data Analysis

Statistical analysis was conducted using R stats v. 2.12.2. Data pertaining to UV and HS optimization were log transformed to meet normal distribution. Analyses of variance (ANOVA) were then conducted on fertilization rate using UV irradiation as independent variable and on diploid gynogen yield with the different HS parameters as independent variables. Values were taken as significant when P < 0.5.

Individual heterozygosity was calculated across all scored loci according to Coulson and co-workers [63] with modification in terms of genotype scoring. In addition to the common genotype scoring of "1" (heterozygous) or "0" (homozygous), an individual homozygous for an allele shared by both parents was scored as "0.5". The difference between control and HS treatment was tested in an additional ANOVA.

## Authors' contributions

ISP conceptualized and designed the experiment, carried out all the experiments, and wrote the manuscript. CE provided the markers for microsatellite analysis, conducted most of the statistical analyses, and helped draft the manuscript. JPS helped in the flow cytometry experiment and in drafting the manuscript. TL and MM participated in conceptualizing and designing the experiment, and in drafting the manuscript. All authors read and approved the final manuscript.
